# The Story of Phœnicia

**Published:** 1889-08

**Authors:** 


**The Story of Phœnicia.** By GEORGE RAWLINSON, M. A. 12mo, pp. 350. New York: G. P. Putnam's Sons. 1889.

The author's name is an indication of the value of this history. It is the twenty-fourth volume of the Story of the Nations series. It contains an excellent description of Phœnicia, its people, their habits, and industries. It is profusely illustrated, and contains a map of the country, which is a great help to all readers of history. It fulfils its mission admirably, in placing the history of Phœnicia before the public in an interesting and attractively readable manner. H. B.

